# PD-L1 blockade in combination with inhibition of MAPK oncogenic signaling in patients with advanced melanoma

**DOI:** 10.1038/s41467-020-19810-w

**Published:** 2020-12-07

**Authors:** Antoni Ribas, Alain Algazi, Paolo A. Ascierto, Marcus O. Butler, Sunandana Chandra, Michael Gordon, Leonel Hernandez-Aya, Donald Lawrence, Jose Lutzky, Wilson H. Miller, Katie M. Campbell, Bruno Delafont, Shannon Marshall, Nancy Mueller, Caroline Robert

**Affiliations:** 1 University of California Los Angeles (UCLA) and Jonsson Comprehensive Cancer Center at UCLA, Los Angeles, CA USA; 2 UCSF Medical Center, San Francisco, CA USA; 3 Istituto Nazionale Tumori IRCCS Fondazione Pascale, Naples, Italy; 4 Princess Margaret Cancer Centre, Toronto, ON Canada; 5 Northwestern Memorial Hospital, Chicago, IL USA; 6 HonorHealth Research Institute, Scottsdale, AZ USA; 7 Washington University School of Medicine, St. Louis, MO USA; 8 Massachusetts General Hospital, Boston, MA USA; 9 Mount Sinai Medical Center, Miami Beach, FL USA; 10 Segal Cancer Center, Jewish General Hospital, Rossy Cancer Network, McGill University, Montreal, QC Canada; 11 AstraZeneca, Gaithersburg, MD USA; 12 Gustave Roussy Cancer Campus and Paris-Saclay University, Villejuif, France

**Keywords:** Cancer, Immunology

## Abstract

Combining PD-L1 blockade with inhibition of oncogenic mitogen-activated protein kinase (MAPK) signaling may result in long-lasting responses in patients with advanced melanoma. This phase 1, open-label, dose-escalation and -expansion study (NCT02027961) investigated safety, tolerability and preliminary efficacy of durvalumab (anti–PD-L1) combined with dabrafenib (BRAF inhibitor) and trametinib (MEK inhibitor) for patients with BRAF-mutated melanoma (cohort A, n = 26), or durvalumab and trametinib given concomitantly (cohort B, *n* = 20) or sequentially (cohort C, n = 22) for patients with *BRAF*-wild type melanoma. Adverse events and treatment discontinuation rates were more common than previously reported for these agents given as monotherapy. Objective responses were observed in 69.2% (cohort A), 20.0% (cohort B) and 31.8% (cohort C) of patients, with evidence of improved tumor immune infiltration and durable responses in a subset of patients with available biopsy samples. In conclusion, combined MAPK inhibition and anti–PD-L1 therapy may provide treatment options for patients with advanced melanoma.

## Abstract (web summary)

Immune checkpoints inhibitors or MAPK inhibitors are currently used as standard of care therapies for patients with advanced melanoma. Here the authors report a phase 1 clinical trial testing the anti-PD-L1 antibody durvalumab in combination with the BRAF inhibitor dafrafenib and the MEK inhibitor trametinib in patients with BRAFV600-mutant melanoma.

## Introduction

The most active standard-of-care therapies for patients with advanced melanoma are divided into two categories, each with its own unique properties and characteristics: (i) small molecule targeted therapy inhibiting aberrant signaling in the mitogen-activated protein kinase (MAPK) pathway, and (ii) immune checkpoint inhibitors, with monoclonal antibodies targeting the programmed death receptor 1 (PD-1) and the cytotoxic T lymphocyte antigen-4 (CTLA-4)^1–3^. However, as a significant proportion of patients do not respond or progress after initial therapy^4–9^, further treatment options are needed for patients with advanced melanoma. Of particular scientific and clinical interest has been combining both modes of therapy, MAPK pathway inhibitors with cancer immunotherapies. This is supported by the knowledge that blocking oncogenic *BRAF*^V600^ signaling results in increased sensitivity of melanoma cells to immunotherapy and increasing antitumor activity in combination with immunotherapy in mouse models^10–12^, while in patients it makes melanoma metastases permissive to immune cell infiltration^13^.

To maximize the clinical benefits of these distinct classes of therapeutics, several studies have explored concurrent and sequential combinations of MAPK inhibitors and immune checkpoint inhibitors in patients with advanced melanoma; many have examined combinations of BRAF and MEK inhibitors with anti-PD-1 or anti-PD-L1 antibodies in patients with *BRAF*^*V600*^ mutant melanoma. A single-arm phase 1b study of the BRAF inhibitor vemurafenib, the MEK inhibitor cobimetinib, and the PD-L1 antibody atezolizumab showed an objective response rate of 71.8% and a median response duration of 17.4 months^14^. Furthermore, a phase 1 trial followed by a randomized phase 2 trial of the BRAF inhibitor dabrafenib and the MEK inhibitor trametinib with or without pembrolizumab demonstrated significant improvement in progression-free survival (PFS) with triple therapy despite an 8.4% lower response rate, and at the expense of increased toxicity^15,16^. Recently, results from the primary analysis of the first randomized phase 3 clinical trial comparing the triple combination of atezolizumab with vemurafenib and cobimetinib, compared to placebo-controlled vemurafenib and cobimetinib, demonstrated a significant improvement in PFS^17^. Patients in the control arm with vemurafenib and cobimetinib double targeted therapy had a median PFS of 10.6 months, which was improved to 15.1 months with the addition of atezolizumab (hazard ratio 0.78)^17^. There was an increase in some toxicities with the triple therapy, in particular, increased creatinine phosphokinase, transaminases, and lipase, as well as an increase in arthralgia and pyrexia, but no change in the rate of discontinuation of study drugs due to toxicities^17^.

Here we report the phase 1 clinical trial testing triple therapy with dabrafenib, trametinib, and the anti-PD-L1 antibody durvalumab in patients with *BRAF*^*V600*^-mutant melanoma with a long-term follow-up (at least 3 years in all patients) to analyze the effect of these therapies on patients. The study includes immune monitoring to explore potential combinatorial effects as well as interference between these therapeutic modalities. In addition, we examine clinical and immunologic effects of concurrent and sequential dosing of trametinib and durvalumab in patients with *BRAF*-wild type melanoma, including those with prior progression on anti-PD-1 antibodies, to explore the potential interactions between these agents in patients in whom immunologic antitumor effects are expected to predominate.

## Results

### Patient characteristics

Between December 2013 and April 2015, 99 patients were screened. Sixty-eight patients were enrolled from 11 study centers (Supplementary Fig. 1, Supplementary Data 1), with 31 patients subsequently excluded due to screening failures. Data cutoff for this analysis was May 30, 2018, when there were at least 3 years of follow-up in all study subjects.

Cohort A initially included six patients who received durvalumab 3 mg/kg every 2 weeks (Q2W) plus standard doses of dabrafenib plus trametinib. Once the safety of this dose was assessed, an additional 20 patients received durvalumab at 10 mg/kg Q2W, of which 7 were enrolled and treated in the second dose-escalation phase and 13 were enrolled and treated as part of the dose-expansion phase. Twenty patients were enrolled in cohort B and received durvalumab 10 mg/kg Q2W concomitantly with trametinib 2 mg every day (QD); 6 patients were enrolled and treated in the dose-escalation phase and 14 patients were enrolled and treated in the dose-expansion phase. In cohort C, 22 patients received the same combination but sequentially (trametinib: days 1–42; durvalumab: from day 29); 7 patients were enrolled and treated in the dose-escalation phase and 15 patients were enrolled and treated in the dose-expansion phase (Supplementary Fig. 1). The median age in cohort A was 49.0 years (range: 23–71) (Table 1), which is consistent with younger age observed in patients with *BRAF*^*V600*^*-*mutant melanoma^7^. Cohorts B and C had a median age of 68.0 years (range: 31–85) and 63.0 years (34–84), respectively (Table 1). The majority of patients—21 (80.8%), 18 (90.0%), and 18 (81.8%) for cohorts A, B, and C, respectively—had stage IV metastatic melanoma. Median lactate dehydrogenase (LDH) levels at baseline for cohorts A, B, and C were 206.5 units per liter (U/L), 215.0 U/L, and 225.0 U/L, respectively; levels were elevated in approximately one-third of patients per cohort (Table 1). In cohort A, 10 (38.5%) patients had received prior systemic therapies, which was a lower proportion than those seen in cohort B (13 [65.0%]) and cohort C (15 [68.2%]). Prior treatments in cohorts B and C included immunotherapy in 12 (60.0%) and 11 (50.0%) patients, respectively.

**Table 1:** Patient demographics at baseline (as-treated population).

Characteristic	Cohort A: Durvalumab 3 or 10 mg/kg + dabrafenib + trametinib (*n* = 26)	Cohort B: Durvalumab 10 mg/kg + trametinib (concurrent) (*n* = 20)	Cohort C: Durvalumab 10 mg/kg + trametinib (sequential) (*n* = 22)
Age, median (range), years	49.0 (23–71)	68.0 (31–85)	63.0 (34–84)
Male sex, *n* (%)	14 (53.8)	13 (65.0)	11 (50.0)
*LDH level*^a^, *n(%)*			
Normal	15 (57.7%)	14 (70.0%)	12 (54.5%)
High	9 (34.6%)	6 (30.0%)	7 (31.8%)
Missing	2 (7.7%)	0	3 (13.6%)
*Race*			
Black or African American	0	0	1 (4.5)
White	20 (76.9)	15 (75.0)	13 (59.1)
Not collected	6 (23.1)	5 (25.0)	6 (27.3)
*Mutation status,n (%)*			
*BRAF*-wild type	0	19 (95.0)	22 (100.0)
*NRAS* mutant	0	4 (20.0)	7 (31.8)
Other mutation	0	1 (5.0)^b^	0
*BRAF*^*V600E*^	19 (73.1)	0	0
*BRAF*^*V600E/K*^	7 (26.9)	0	0
*Stage at study entry,n(%)*			
III	5 (19.2)	2 (10.0)	4 (18.2)
IV	21 (80.8)	18 (90.0)	18 (81.8)
*Metastasis stage at initial diagnosis, n (%)*			
M0	14 (53.8)	10 (50)	14 (63.6)
M1	0	1 (5)	0
M1A	2 (7.7)	0	1 (4.5)
M1B	0	1 (5)	0
M1C	3 11.5)	1 (5)	1 (4.5)
MX	0	2 (10)	1 (4.5)
Unknown	7 (26.9)	5 (25)	5 (22.7)
*Baseline ECOG performance status, n (%)*			
0	19 (73.1)	13 (65.0)	12 (54.5)
1	5 (19.2)	7 (35.0)	9 (40.9)
Missing	2 (7.7)	0	1 (4.5)
*Number of prior systemic regimens, n (%)*			
0	15 (57.7)	6 (30.0)	7 (31.8)
1	5 (19.2)	2 (10.0)	8 (36.4)
2	5 (19.2)	4 (20.0)	1 (4.5)
3	0	3 (15.0)	4 (18.2)
≥4	0	4 (20.0)	2 (9.1)
Unknown	1 (3.8)	1 (5.0)	0
*Patients with prior immunotherapy in adjuvant or metastatic setting, n (%)*			
Any	10 (38.5)	12 (60.0)	11 (50.0)
Anti-CTLA-4 inhibitor	6 (23.1)	10 (50.0)	7 (31.8)
Anti-PD-1 or anti-PD-L1	0	6 (30.0)	5 (22.7)
Cytokine-based therapy	7 (26.9)	6 (30.0)	6 (27.3)

^a^Normal LDH levels range from 140 to 280 U/L)^37^.

^b^Patient was positive for BRAF Q456H & G464A.

*CTLA-4* cytotoxic T-lymphocyte-associated antigen 4, *ECOG* Eastern Cooperative Oncology Group, *LDH* lactic acid dehydrogenase, *PD-1* programmed cell death protein-1, *PD-L1* programmed cell death-ligand 1.

### Patient dispositions

The median treatment duration was 10.4 months in cohort A, 6.3 months in cohort B, and 5.9 months in cohort C. Eleven (42.3%), 6 (30.0%), and 5 (22.7%) patients, respectively, completed the intended 12 months of durvalumab treatment, and were eligible to continue with the targeted therapy beyond that time. For those who did not complete durvalumab treatment, the most common reasons for treatment discontinuation were disease progression, occurring in 8 (30.8%), 9 (45.0%), and 11 (50.0%) patients in cohorts A, B, and C, respectively, with adverse events (AEs), occurring in 5 (19.2%), 4 (20.0%), and 3 (13.6%) patients, respectively. Following the protocol-specified option to be treated beyond progression and receive a new cycle of durvalumab therapy, 8 patients received retreatment with durvalumab, 3 of whom completed an additional 12 months of treatment.

### Safety and tolerability

The most common treatment-emergent AEs deemed related to any of the study drugs investigated are listed in Supplementary Table 1. The most common treatment-related AEs in cohort A were pyrexia (76.9%), chills (65.4%), fatigue (61.5%), and arthralgia (50.0%); the majority of which were grade 1/2 (2 and 1 patients [both received 10 mg/kg durvalumab] reported grade 3 treatment-related pyrexia and arthralgia, respectively). The most common treatment-related AEs in cohorts B and C, respectively, were diarrhea (55.0% and 40.9%) and rash (35.0 and 50.0%); the majority of which were grade 1/2 (1 and 2 patients [both cohort C] reported grade 3 treatment-related diarrhea and rash, respectively. None of the patients in cohort A, 1 patient (5.0%) in cohort B, and 1 patient (4.5%) in cohort C had an increase in liver enzymes. Immune-related AEs were reported in all study cohorts, including hyperthyroidism in 1 patient (3.8%) in cohort A, grade 2 pneumonitis in 1 (5.0%) in cohort B, and autoimmune hepatitis in 1 (4.5%) in cohort C.

Grade ≥3 AEs were reported in 18 (69.2%) patients in cohort A (3 patients received 3 mg/kg durvalumab and 15 patients received the 10 mg/kg dose), 16 (80.0%) in cohort B, and 16 (72.7%) in cohort C (Table 2). There was no consistent difference in tolerability or toxicities between the patients in cohort A who received durvalumab at 3 or 10 mg/kg (Table 2). Serious AEs were reported in 16 (61.5% [2 patients received 3 mg/kg durvalumab and 14 patients received 10 mg/kg durvalumab]), 9 (45.0%), and 10 (45.5%), respectively. AEs that led to dose modifications or discontinuations, respectively, following any of the study drugs were reported in 23 (88.5% [5 and 18 patients from the 3 and 10 mg/kg groups, respectively]) and 12 (46.2%) patients in cohort A, 20 (100%) and 7 (35.0%) patients in cohort B, and 16 (72.7%) and 11 (50.0%) patients in cohort C. The most common reasons for treatment discontinuation due to treatment-emergent AEs were pyrexia (4% of all patients, *n* = 3), blood creatinine phosphokinase increase (3%, *n* = 2), and ejection fraction decrease (3%, *n* = 2). No deaths related to the study drugs and no on-treatment deaths occurred.

**Table 2:** Summary of treatment-emergent adverse events (as-treated population).

Adverse event, *n* (%)^a^	Cohort A: Durvalumab 3 mg/kg + dabrafenib + trametinib (*n* = 6)	Cohort A: Durvalumab 10 mg/kg + dabrafenib + trametinib (*n* = 20)	Cohort B: Durvalumab 10 mg/kg + trametinib (concurrent) (*n* = 20)	Cohort C: Durvalumab 10 mg/kg + trametinib (sequential) (*n* = 20)
≥1 event of ≥ grade 3 severity^b^	3 (50.0)	15 (75.0)	16 (80.0)	16 (72.7)
Deaths	0	6 (30.0)	4 (20.0)	1 (4.5)
≥1 serious event^c^	2 (33.3)	14 (70.0)	9 (45.0)	10 (45.5)
≥1 event leading to discontinuation of any of the study products	4 (66.7)	8 (40.0)	7 (35.0)	11 (50.0)
≥1 event leading to dose modification of any of the study products^d^	5 (83.3)	18 (90.0)	20 (100)	16 (72.7)

^a^Patients are counted once for each category, regardless of the number of events.

^b^Grade 3 = severe; grade 4 = life-threatening.

^c^Serious adverse event criteria: death, life-threatening event, required inpatient hospitalization, prolongation of existing hospitalization, persistent or significant disability/incapacity, important medical event, or congenital anomaly/birth defect in offspring.

^d^Dose modification includes any dose reduction, delay, interruption, or permanent discontinuation.

No clinically meaningful trends in laboratory parameters or electrocardiogram (ECG) findings were observed.

### Efficacy

**Fig. 1:**
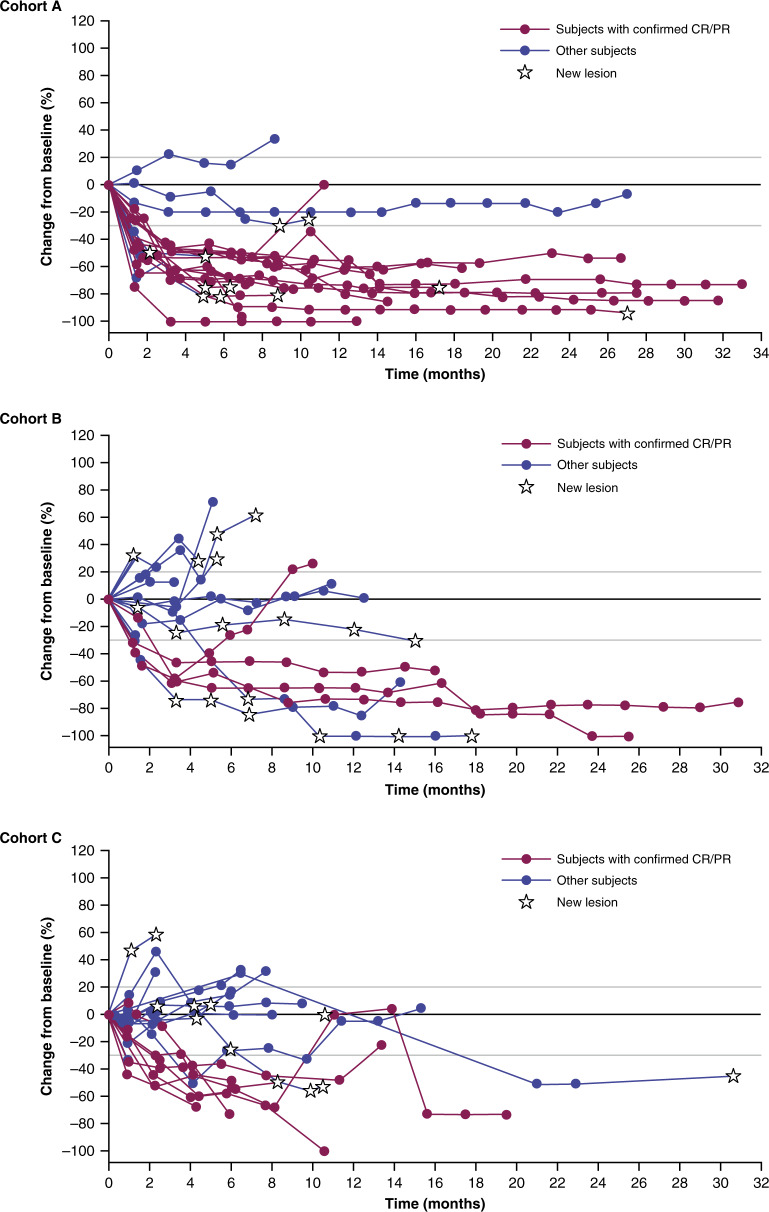
Best percentage change from baseline in tumor diameter (as-treated population). Longitudinal tumor size was analyzed using a non-linear mixed-effects model to determine tumor growth rate constants and time to growth. Cohort A, *n* = 26; Cohort B, *n* = 20; Cohort C, *n* = 22. CR/PR, complete response/partial response.

**Fig. 2:**
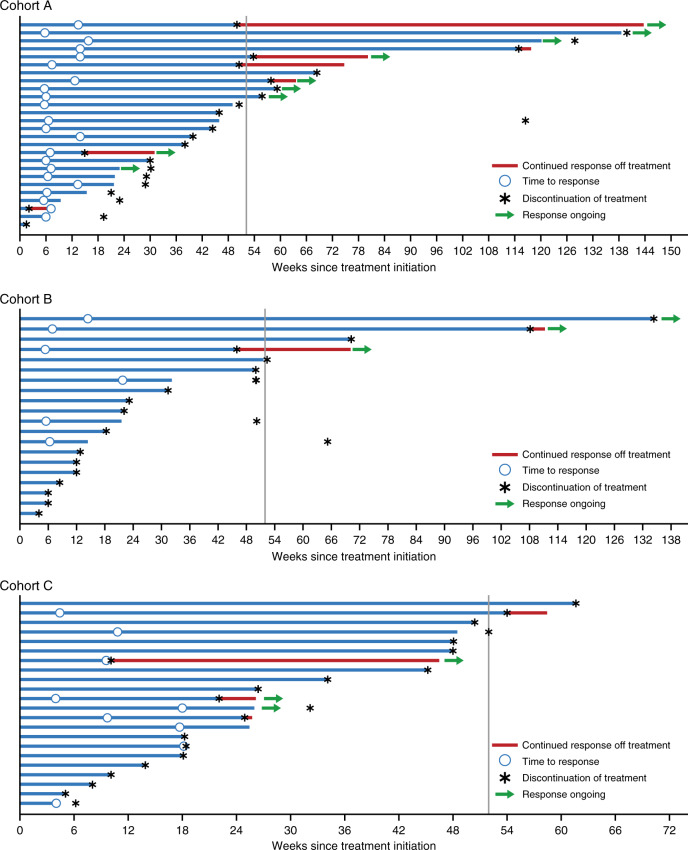
Duration of response (as-treated population). Vertical lines indicate planned 12 months of treatment with durvalumab. Cohort A, *n* = 26; Cohort B, *n* = 20; Cohort C, *n* = 22.

In cohort A, the median duration of follow-up was 20.8 months (range: 3.4–38.7). The overall response rate (ORR) was 69.2% (95% CI: 48.2–85.7), corresponding to 18 patients (Table 3). The median best percentage change from baseline in target lesion diameter was −70.6% (range: −100.0 to +10.1) (Fig. 1). Of the 18 patients who had an objective response, 9 (50.0%) had ongoing responses as of the data cutoff, with a median duration of response of 15.5 months (Fig. 2). The median PFS was 11.2 months (95% CI: 8.9–NA), and the median overall survival (OS) was 31.4 months (95% CI: 14.3–NA) (Table 3; Supplementary Fig. 2). The OS rate at 12 months was 76% (95% CI: 54–88).

**Table 3:** Clinical activity (as-treated population).

Response	Cohort A: Durvalumab 3 or 10 mg/kg + dabrafenib + trametinib (*n* = 26)	Cohort B: Durvalumab 10 mg/kg + trametinib (concurrent) (*n* = 20)	Cohort C: Durvalumab 10 mg/kg + trametinib (sequential) (*n* = 22)
ORR (CR + PR), *n* (%)	18 (69.2)	4 (20.0)	7 (31.8)
DCR (CR + PR + SD), *n* (%)	25 (96.2)	16 (80.0)	14 (63.6)
CR, *n* (%)	3 (11.5)	1 (5.0)	0
PR, *n* (%)	15 (57.7)	3 (15.0)	7 (31.8)
SD, *n* (%)	7 (26.9)	12 (60.0)	7 (31.8)
DCR12, *n* (%)	23 (88.5)	14 (70.0)	13 (59.1)
Median DoR, months	15.5	NA	8.7
Median PFS (95% CI), months	11.2 (8.9–NA)	4.9 (3.0–5.5)	5.9 (2.4–11.1)
6-month PFS rate (95% CI), %	78.3 (55.4–90.3)	28.2 (10.3–49.4)	41.3 (18.3–63.1)
12-month PFS rate (95% CI), %	49.1 (27.0–68.0)	22.6 (7.0–43.4)	15.5 (1.2–45.4)
Median OS (95% CI), months	31.4 (14.3–NA)	NA (6.9–NA)	21.7 (12.0–NA)
12-month OS rate (95% CI), %	76 (54–88)	63 (38–80)	70 (46–85)

*CR* complete response, *DCR* disease control rate, *DCR12* disease control rate at 12 weeks, *DoR* duration of response, *NA* not available, *ORR* overall response rate, *OS* overall survival, *PFS* progression-free survival, *PR* partial response, *SD* stable disease.

The median duration of follow-up was 22.1 months (range: 1.7–34.0) months in cohort B and 20.8 months (range: 1.1–36.1) in cohort C. The ORRs were 20.0% (95% CI: 5.7–43.7) in cohort B and 31.8% (95% CI: 13.9–54.9) in cohort C, corresponding to 4 and 7 patients respectively (Table 3). Among the 11 patients with *NRAS*-mutant disease across cohorts B and C, 3 (27.2%) had PRs. The median best percentage change from baseline in target lesion diameter was −13.4% (range: −100 to +32.0) in cohort B and −33.5% (range: −100 to +46.6) in cohort C (Fig. 1). Of the 4 patients in cohort B and 7 patients in cohort C who had a response, 3 from each cohort (75.0% and 42.9%, respectively), continued to respond at the data cutoff; the median duration of response could not be calculated in cohort B (response ongoing for most patients), and was 8.7 months in cohort C. The median PFS was 4.9 months (95% CI: 3.0–5.5) in cohort B and 5.9 months (95% CI: 2.4–11.1) in cohort C. The median OS was not reached in cohort B and was 21.7 months (95% CI: 12.0–NA) in cohort C (Table 3; Supplementary Fig. 2). The OS rate at 12 months was 63% (95% CI: 38–80) in cohort B and 70% (95% CI: 46–85) in cohort C.

### Pharmacokinetic analyses

Pharmacokinetic (PK) data were available for all 68 patients. PK exposure of durvalumab was comparable across the different combination arms with 10 mg/kg Q2W durvalumab (Supplementary Table 3), suggesting combination with dabrafenib and/or trametinib did not impact durvalumab exposure in patients.

In general, PK exposure of dabrafenib and dabrafenib metabolites were maintained over the time course of dabrafenib treatment, with comparable geometric means of pre-dose concentrations of dabrafenib observed across study days and treatment combinations (Supplementary Table 4).

PK exposure of trametinib was maintained over the time course of trametinib treatment and comparable in each cohort. Across all cohorts, geometric means of pre-dose concentrations of trametinib were comparable on the same study day (Supplementary Table 5), suggesting that combining with different durvalumab doses and dabrafenib did not impact PK exposure of trametinib.

As a whole, PK concentrations of durvalumab, dabrafenib, and trametinib were consistent with PK observations from previous studies using similar dosing regimens^18–22^.

### Immunogenicity

Immunogenicity data were available for a total of 66 of 68 patients. Of the 66 patients tested, all were negative for ADAs to durvalumab at baseline. Five (7.6%) patients tested positive for treatment-emergent durvalumab ADAs; all 5 patients were transiently positive for durvalumab ADAs. Overall, PK exposure of durvalumab was similar between ADA treatment-emergent positive and negative patients, indicating that the effect of immunogenicity on PK exposure of durvalumab was minimal. No clear evidence of any potential impact of ADAs on safety were observed, with the AEs reported in ADA-positive patients similar to those reported in patients who were ADA-negative.

### Pharmacodynamic analyses

**Fig. 3:**
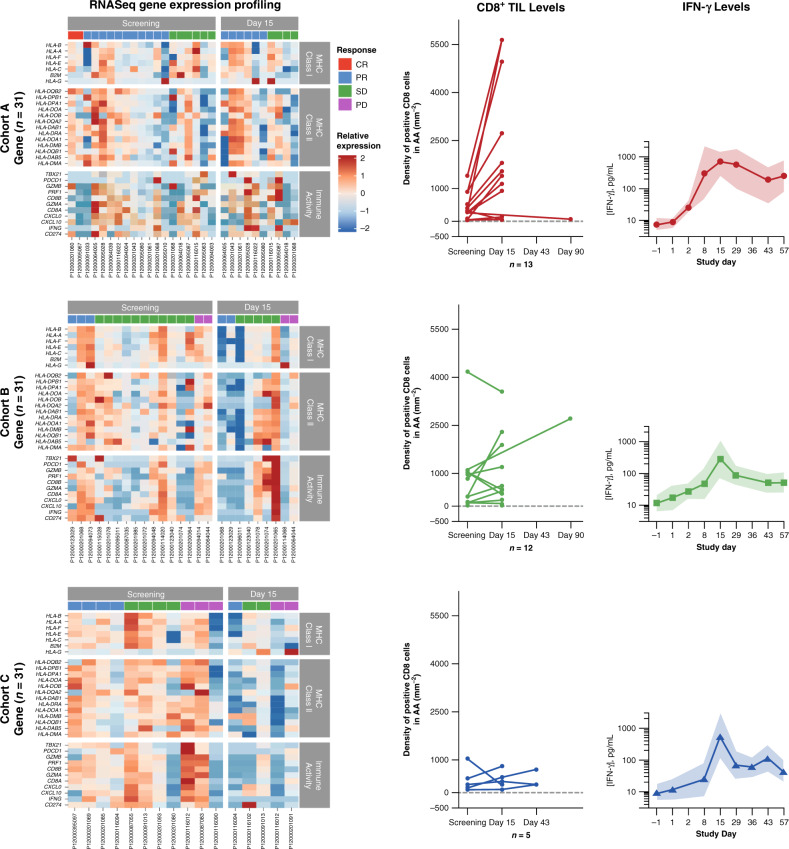
RNA-sequenced gene expression profiling, CD8+ T-cell infiltration by IHC, and IFNγ levels in circulating blood. Cohort A, *n* = 13; Cohort B, *n* = 12; Cohort C, *n* = 5. CR complete response, IHC immunohistochemistry, IFN-γ interferon-gamma, PR partial response, PD progressive disease, SD stable disease, TIL tumor-infiltrating lymphocyte.

Biopsies for analysis of changes in CD8+ T-cell infiltration by immunohistochemistry were available from 13 patients in cohort A, 12 in cohort B, and 5 in cohort C (Fig. 3). Baseline CD8+ cell infiltration was higher in cohort A (median density 432.0 cells/mm^2^) than in cohort C (388.2 cells/mm^2^), and highest in cohort B (median 835.5 cells/mm^2^); the difference may have been influenced by patients in cohort A being less likely to be pretreated for metastatic melanoma. Changes in immune infiltration were common in cohort A: CD8+ cell density increased in 9 of 13 biopsies at day 15. Changes in CD8+ cell density were not as consistent in biopsies of patients from cohorts B and C. Because patients in cohort C received sequential therapy with trametinib and durvalumab, with a partial overlap over 2 weeks, we analyzed CD8+ cell infiltration on day 15 when they were on trametinib alone and on day 43 when they were receiving the combination. However, there was no evidence of change in CD8+ cell density in the 3 patients that had sequential biopsies for analyses at all 3 time points (baseline, day 15, and day 43). Although the study was not powered to make a direct comparison, these data may suggest a more frequent increase in CD8+ cell infiltration in patients with *BRAF*-mutated melanoma receiving BRAF plus MEK inhibitors with anti-PD-L1 therapy than in patients with *BRAF*-wild type melanoma receiving the MEK inhibitor with anti-PD-L1 therapy.

RNA-sequenced gene expression profiling was performed on 95 biopsies from 65 patients; of these, 84 samples had adequate quality for analysis (Supplementary Fig. 3, Supplementary Table 2). The analyzed biopsies were from 20 patients in cohort A (including 13 with CRs or PRs, 6 with stable disease [SD], and 1 not evaluated), 16 patients in cohort B (including 3 with PRs, 11 with SD, and 2 with disease progression), and 14 patients in cohort C (including 4 with PRs, 4 with SD, 3 with disease progression, and 3 not evaluated) (Fig. 3, Supplementary Fig. 4).

In cohort A, objective responses were observed regardless of baseline immune gene expression, suggesting that response to this triple therapy combination is not limited to patients with a pre-existing immune infiltrate, as is the case with single-agent PD-1/L1 blockade therapy^23^. Biopsies on day 15 from patients in cohort A who had a response to therapy had higher levels of immune gene expression than those from patients with SD, but there was wide variability among the biopsies. Similar variability in immune gene expression was noted in biopsies from cohorts B and C, with an evident decrease from baseline in expression in the on-therapy biopsies of cohort C, which were taken at the time that patients received trametinib alone and were the only group of on-therapy biopsies while patients were not receiving durvalumab. These data suggest that single-agent therapy with trametinib in cohort C resulted in an apparent decrease in immune gene expression in biopsies compared with combination therapy including durvalumab in the two other cohorts.

We also analyzed interferon-gamma (IFNγ) levels in circulating blood from patients in all 3 cohorts (Fig. 3). The triple therapy used in cohort A resulted in a rapid increase that peaked on day 15 and remained high in subsequent blood samples up to day 57. In contrast, cohorts B and C had slower increases in circulating blood IFNγ levels, which also peaked at day 15 but were lower in subsequent blood samples. These data suggest that systemic immune activation occurred in all cohorts, but was more pronounced in cohort A.

## Discussion

This multicohort phase 1 clinical trial provides confirmation of the combinatorial effects between BRAF plus MEK inhibitor therapy and anti-PD-L1 blockade in BRAF-mutated melanoma, including patients who had previously progressed on anti-CTLA-4 therapy, with supporting data on the immune-stimulatory effects of the triple therapy combination provided by RNA sequencing. We also tested the combination of a MEK inhibitor with anti-PD-L1 antibody therapy, both concomitant and sequentially.

The immune monitoring data for the triplet combination of dabrafenib, trametinib, and durvalumab provide continued support for the concept of a favorable immunologic interaction between MAPK inhibitors and anti-PD-1 antibodies. As reported in previous studies^14,16^, the triplet combination was associated with increases in CD8+ T-cell density, which is associated with productive antitumor immune responses. Furthermore, objective responses were seen even in patients without substantial baseline CD8+ T-cell infiltrate, a population that might be unresponsive to anti-PD-1 antibody monotherapy; increased circulating IFNγ levels were observed in cohort A overall. Previous work supports an independent role of BRAF and MEK inhibitor combination therapy in increasing tumor-infiltrating lymphocyte populations and inflammatory cytokine levels, suggesting that this effect cannot be attributed solely to exposure to the anti-PD-L1 antibody, durvalumab^10,14^. Although our sample size was small, half of the patients receiving triplet therapy remained in remission at a median follow-up of 20.8 months, which compares favorably to historical controls for combined BRAF plus MEK inhibitors^24–26^. However, in the absence of a durvalumab monotherapy lead-in and paired biopsies, the immunologic contribution of the PD-L1 inhibitor to long-term patient benefits could not be isolated.

In cohort C of the current study, in which trametinib was initially given alone, there was no increase in tumor-infiltrating CD8+ T cells in three patients with available paired tumor biopsies. In addition, inflammatory gene expression actually decreased in patients treated with trametinib alone, while this effect was not seen in patients treated with concurrent trametinib and durvalumab. Although these data are preliminary given the small sample size, they suggest that trametinib could have an immune inhibitory effect that is offset by the addition of durvalumab. In addition, circulating IFNγ levels appeared to increase more gradually and in a less sustained manner in patients in cohorts B and C, compared with patients in cohort A with increased MAPK inhibitor-sensitive *BRAF*-mutant melanoma, even though the BRAF plus MEK inhibitor combination is expected to have less impact on wild type cells than the MEK inhibitor alone; a recognized paradoxical effect^27^.

Overall, the clinical response data for the trametinib plus durvalumab combination arms in *BRAF*-wild type melanoma could not clearly demonstrate whether doublet therapy improved the response rate in comparison with historical controls. In cohort B, in which trametinib and durvalumab were administered concurrently from the outset, the ORR was 20.0%, and in cohort C, in which the agents were initiated sequentially, the ORR was 31.8%. These estimates in relatively small patient samples are similar to, if not slightly lower, than those observed in patients treated with anti-PD-1 antibody monotherapy^2,28,29^, which may reflect that this study allowed inclusion of patients who had prior progression to PD-1 blockade therapy. Of the patients in cohorts B and C, 30% were previously treated and progressed on anti-PD-1 antibodies. One of these 11 patients responded to combination therapy and 9 patients had SD; it remains unclear whether the addition of MEK inhibition could promote antitumor immunity and response to anti-PD-1 and anti-PD-L1 antibodies in a selected subset of patients.

In summary, the triplet combination of dabrafenib, trametinib, and durvalumab is feasible in patients with *BRAF*-mutant advanced melanoma and induces robust and sustained immune modulation. The increase in toxicities observed with the combination of the three therapies needs to be evaluated within the context of the limited benefits observed when targeted therapies or immunotherapy treatments are given separately in those patients with the most aggressive disease at diagnosis. Additional studies are required to define the role of combined MAPK inhibition and immune checkpoint blockade in patients with advanced melanoma.

## Methods

### Study design and treatment

This was an open-label, dose-escalation, and -expansion study in patients with *BRAF*^*V600*^*-*mutant or *BRAF*-wild type metastatic or unresectable melanoma (NCT02027961). Patients were randomized to receive either durvalumab in combination with dabrafenib and trametinib or with trametinib alone. The study protocol can be found in the Supplementary Notes; Section 4.5 of the study protocol detailing the formulation of durvalumab has been redacted due to legal/intellectual property requirements set by the manufacturer.

Following a 28-day screening phase, patients were enrolled in one of three study cohorts (Supplementary Fig. 5). Cohort A included patients with *BRAF*^*V600E*^- or *BRAF*^*V600K*^-mutant disease who received dabrafenib 150 mg twice daily, trametinib 2 mg once daily, and durvalumab 3 mg/kg (first dose-escalation cohort) or 10 mg/kg administered intravenously Q2W (dose-expansion phase). Dabrafenib and trametinib were administered until confirmed progressive disease or intolerable toxicity, and durvalumab was administered for up to 12 months. Cohort B included patients with *BRAF*-wild type melanoma who received concurrent trametinib 2 mg once daily and durvalumab 10 mg/kg intravenously Q2W for up to 12 months until confirmed disease progression or intolerable toxicity. Cohort C included patients with *BRAF*-wild type melanoma who received sequential doses of trametinib 2 mg once daily for 42 days and durvalumab 10 mg/kg Q2W starting on day 29 for up to 12 months. In all 3 treatment cohorts, durvalumab would be stopped at 12 months in the absence of confirmed progression, initiation of alternative cancer therapy, unacceptable toxicity, withdrawal of consent, or other reason to discontinue treatment.

The study protocol allowed to continue patients on therapy beyond initial progression. In Cohorts A and B, if there was evidence of progression during the post-durvalumab treatment period, durvalumab may be re-administered as an IV infusion Q2W for up to an additional 12 months while continuing dabrafenib and trametinib (Cohort A) or trametinib alone (Cohort B) provided the subject meets the criteria for re-administration in the setting of PD. In Cohort C, durvalumab may be re-administered as an IV infusion Q2W for up to an additional 12 months without concurrent trametinib provided the subject meets the criteria for re-administration in the setting of progressive disease. All subjects will be followed indefinitely for survival, until the sponsor closes the study, or for the maximum duration per institutional standards.

### Patients

Patients with stage IIIC/IV melanoma were eligible for inclusion. To further meet inclusion criteria, patients were required to have ≥1 measurable lesion per Response Evaluation Criteria in Solid Tumors (RECIST) version 1.1^30^, an Eastern Cooperative Oncology Group performance status of 0 or 1, and adequate bone marrow and organ function. Prior immunotherapy with anti-CTLA-4 or anti-PD-1/PD-L1 agent was permitted. Key exclusion criteria included another malignancy within 5 years of study enrollment, active or prior autoimmune disease, prior BRAF or MEK inhibitor therapy, prior severe or persistent immune-related AEs, and a history of primary immunodeficiency. All patients provided written informed consent.

The first patient was enrolled on 20 December 2013 and the last visit for the final patient was on 24 April 2018. Data cutoff for this analysis was May 30, 2018, when there was at least 3 years of follow-up in all study subjects.

### Outcomes

The primary endpoints of safety assessment and determination of the maximum tolerated dose (MTD) included evaluation of dose-limiting toxicities (DLTs, AEs, serious AEs, laboratory evaluations, vital signs, physical examinations, and ECG results. Secondary endpoints were objective response and disease control per RECIST v1.1, duration of response, PFS, OS, pharmacokinetics (C_max_ and C_trough_ after the first and steady-state doses), and immunogenicity (number and percentage of subjects who develop detectable antidrug antibodies [ADAs]).

Exploratory endpoints included antitumor activity as assessed by immune-related RECIST (irRECIST)^31^ during central review of scans, immunohistochemical assessment of PD-L1 expression within the tumor microenvironment and correlation with response to treatment, tumor growth parameters, and pharmacogenomic analysis of blood and tumor samples to identify a gene signature predictive of response. We focused the analysis on the change in expression of genes involved in MHC class I or II, and immune genes related to CD8 and interferon-gamma (IFNγ) signaling, which have been previously reported to increase in tumors from preclinical models testing the combination of BRAF and MEK inhibitors or MEK inhibitors together with immunotherapy^31–33^.

### Procedures

The population for efficacy analysis and safety evaluation included all patients who received any dose of study product (as-treated population). The PK-evaluable population consisted of all subjects who received at least 1 dose of investigational product and at least 1 post-dose evaluable PK result. Immunogenicity data were analyzed in all patients who had a non-missing baseline ADA result and at least one non-missing post-baseline ADA result.

AEs were graded per Common Terminology Criteria for Adverse Events version 4.03 and were assessed throughout the study and for 90 days after the end of treatment. Tumor assessments for the secondary (RECIST v1.1) or exploratory (irRECIST) endpoints were conducted during screening, and throughout the study period until disease progression, death, or withdrawal from the study. In addition to tumor assessments during screening, and throughout the study period, assessment for patients discontinuing all study products due to confirmed disease progression, disease evaluation was performed at the end-of-treatment visit and 30 days thereafter. For patients entering follow-up after discontinuation of all study products due to toxicity, disease evaluation was performed at the end of treatment, every 2 months for 1 year, and then every 6 months thereafter until confirmed disease progression or the end of the study. The response rate was defined as the percentage of patients with a confirmed complete response (CR) or partial response (PR) per RECIST v1.1, as assessed by the site investigators. The best percentage change from baseline in tumor diameter was defined as the maximum reduction from baseline or the minimum increase from baseline in the absence of a reduction. Measurement of durvalumab concentrations in serum and of dabrafenib, dabrafenib metabolites (hydroxy- and desmethyl-dabrafenib), and trametinib concentrations in plasma were performed using a validated immunoassay. Blood samples were collected on day 1, day 8, day 15, and day 29 (±1 day). Presence of ADA will be assessed in samples taken on day 1 and day 29 (±1 day). Samples will be measured for the presence of ADA by the sponsor using a validated bridging immunoassay. For the translational analyses, tumor biopsies were collected at baseline, and then on day 15 (±3 days) and day 43 (±3 days) of the treatment period. For the RNA sequencing (RNAseq), total RNA from each sample was prepared for sequencing using the Takara Bio SMART-Seq: SMART-Seq^®^ v4 Ultra^®^ Low Input RNA Kit. Between 0.8 and 1.3 ng of RNA was used to prepare the RNA libraries. Eleven cycles of PCR were performed during cDNA amplification. Samples were then processed with the Nextera XT DNA sample preparation kits for Illumina. The cDNA was normalized to the modified recommended input amount of 100–150 pg and ten cycles during Nextera library prep. The purified amplified libraries were then validated by Agilent High Sensitivity DNA chip on Agilent 2100 Bioanalyzer and quantitated via qPCR using KAPA Library Quantification Kit (KAPA Biosystems) according to manufacturer’s instructions. The libraries were sequenced on Illumina HiSeq 2500 with the following run parameters: Paired-End/Dual-Indexed 2 × 75 bp reads. RNAseq data were aligned to the human reference genome (GRCh38) by Hisat2^34^. Gene expression was annotated using Ensembl (release 94) and summarized by HTSeq-counts^35^. Gene expression values were normalized and compared across groups using the DESeq2 R package^24^. Gene expression was displayed as the z-score of the normalized gene expression using the ggplot2 R package^36^.

### Statistical design and analysis

The dose-escalation phase followed a standard 3 + 3 design. Where ≤2 DLTs were observed in the dose-escalation phase for each cohort, the dose for durvalumab could be increased to 10 mg/kg (MTD) as part of the dose-expansion phase. The dose levels for the dose escalation of durvalumab in combination with dabrafenib and trametinib in cohort A was based on the dose-escalation safety and pharmacokinetic information using single-agent durvalumab. Based on the prior experience, durvalumab was started at 3 mg/kg Q2W, one dose level below the recommended phase 2 dosing of durvalumab alone, with the next cohort using the full dose of durvalumab at 10 mg/kg Q2W followed by a dose-expansion cohort. The 10 mg/kg is the FDA-approved dose for durvalumab. The primary justification of the schedule for cohort C was the anticipated increase in toxicities with the combination of a MEK inhibitor with an anti-PD-L1 therapy. At the time of starting this study, it was already clear that the combination of a BRAF and MEK inhibitor was less toxic than using either agent alone, given that both drugs offset the toxicities of each other through the phenomenon of paradoxical MAPK activation with a BRAF inhibitor. In particular, MEK inhibitors given as single agent had near-universal acneiform skin rash that limited continuous therapy administration, which was felt could be worsened with the addition of anti-PD-L1.

Approximately 69 patients were required to be enrolled for both the dose-escalation phase and the dose-expansion phase of the study. For the dose-escalation phase, up to 24 evaluable patients were required, with 2 dose levels in cohort A and 1 dose level each in cohorts B and C. For the dose-expansion phase, a minimum of 42 patients were required in the 3 cohorts (~14 patients in each of Cohorts A, B, and C) to ensure ~20 patients were treated at the MTD or 10 mg/kg dose level selected for each cohort. The sample size was mainly chosen to obtain preliminary assessment of antitumor activity (ORR) for the *BRAF*-wild type and *BRAF* mutation-positive cohorts.

Unless otherwise stated, patient dispositions, baseline characteristics, and safety and efficacy analyses were based on the as-treated population, which included all subjects who received any investigational product. Categorical data were summarized by frequency distribution (number and percentage of patients falling within each category). Continuous variables were summarized by descriptive statistics, including N values, means, standard deviations, medians, and ranges. Confidence intervals, whenever specified, were two-sided and produced at 95%. The Kaplan–Meier method was used to estimate PFS and OS, where PFS was defined as the time from the start of treatment with a study product to the first documentation of disease progression or death, whichever came first, and OS was measured from the start of treatment until death. Individual durvalumab, dabrafenib, dabrafenib metabolites, and trametinib concentrations were tabulated by dose cohort, visit, and time points, along with descriptive statistics. For durvalumab, PK parameters were estimated using a non-compartmental PK analysis approach for each dose cohort. Immunogenicity was assessed by summarizing the number and percentage of subjects who develop detectable ADAs.

### Study oversight

The study was performed in accordance with the Declaration of Helsinki and the principles of the International Conference on Harmonisation/Good Clinical Practice and/or local regulatory requirements. The study protocol was approved by the relevant institutional review board/independent ethics committee (see Supplementary Table 6), and AstraZeneca provided regulatory authorities, the institutional review board/independent ethics committee, and principal investigators with safety updates/reports. Dose-escalation and dose-expansion decisions were made by a study-specific committee.

### Reporting summary

Further information on research design is available in the Nature Research Reporting Summary linked to this article.

## Supplementary information

Supplementary Figures and Tables

Reporting Summary

Supplementary Data 1

Description of Additional Supplementary File

## Data Availability

The clinical dataset analyzed here is available and may be obtained in accordance with AstraZeneca’s data sharing policy, which is described at https://astrazenecagrouptrials.pharmacm.com/ST/Submission/Disclosure. RNAseq data is available in GEO under GSE158403.
