# Author Correction: PD-L1 blockade in combination with inhibition of MAPK oncogenic signaling in patients with advanced melanoma

**DOI:** 10.1038/s41467-021-25285-0

**Published:** 2021-08-12

**Authors:** Antoni Ribas, Alain Algazi, Paolo A. Ascierto, Marcus O. Butler, Sunandana Chandra, Michael Gordon, Leonel Hernandez-Aya, Donald Lawrence, Jose Lutzky, Wilson H. Miller, Katie M. Campbell, Bruno Delafont, Shannon Marshall, Nancy Mueller, Caroline Robert

**Affiliations:** 1 University of California Los Angeles (UCLA) and Jonsson Comprehensive Cancer Center at UCLA, Los Angeles, CA USA; 2 UCSF Medical Center, San Francisco, CA USA; 3 Istituto Nazionale Tumori IRCCS Fondazione Pascale, Naples, Italy; 4 Princess Margaret Cancer Centre, Toronto, ON Canada; 5 Northwestern Memorial Hospital, Chicago, IL USA; 6 HonorHealth Research Institute, Scottsdale, AZ USA; 7 Washington University School of Medicine, St. Louis, MO USA; 8 Massachusetts General Hospital, Boston, MA USA; 9 Mount Sinai Medical Center, Miami Beach, FL USA; 10 Segal Cancer Center, Jewish General Hospital, Rossy Cancer Network, McGill University, Montreal, QC Canada; 11 AstraZeneca, Gaithersburg, MD USA; 12 Gustave Roussy Cancer Campus and Paris-Saclay University, Villejuif, France

**Keywords:** Cancer, Immunology

Correction to: *Nature Communications* 10.1038/s41467-020-19810-w, published online 7 December 2020

There was an error in the original version of this Article. A table describing the patient characteristics and the sample collection was not included in the paper. This table is now included as Supplementary Data 1 and the first sentence of the Results section has been amended to state ‘Between December 2013 and April 2015, 99 patients were screened. Sixty-eight patients were enrolled from 11 study centers (Supplementary Fig. 1, Supplementary Data 1), with 31 patients subsequently excluded due to screening failures’. The PDF and HTML versions of the Article have been updated.

## Supplementary information

Description of Additional Supplementary File

Supplementary Data 1

