# Supplementary material for: PD-L1 blockade in combination with inhibition of MAPK oncogenic signaling in patients with advanced melanoma
Source: Nat Commun. 2020 Dec 7;11:6262. doi: 10.1038/s41467-020-19810-w (PMC7721806; doi:10.1038/s41467-020-19810-w)
Supplement: Supplementary file 1 — Supplementary Figures and Tables [file 41467_2020_19810_MOESM1_ESM.pdf]

SUPPLEMENTARY DATA

Ribas et al. PD-L1 blockade in combination with inhibition of MAPK oncogenic signaling in patients with advanced melanoma.

E-mail: aribas@mednet.ucla.edu

Supplementary Figures

Supplementary figure 1. CONSORT diagram.

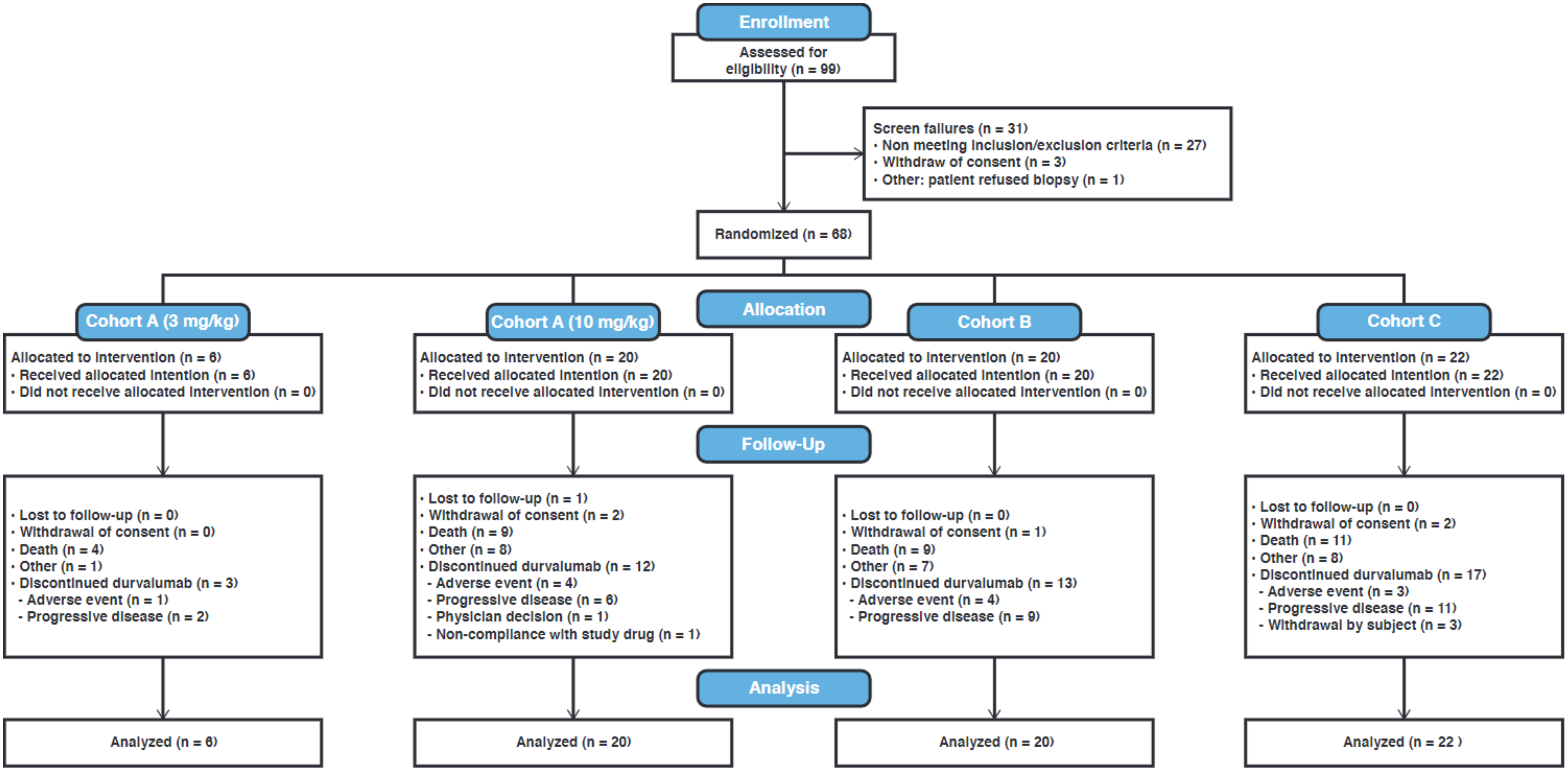

Supplementary figure 2. Kaplan-Meier plots of OS (a) and PFS (b) for cohorts A, B, and C (as-treated population)

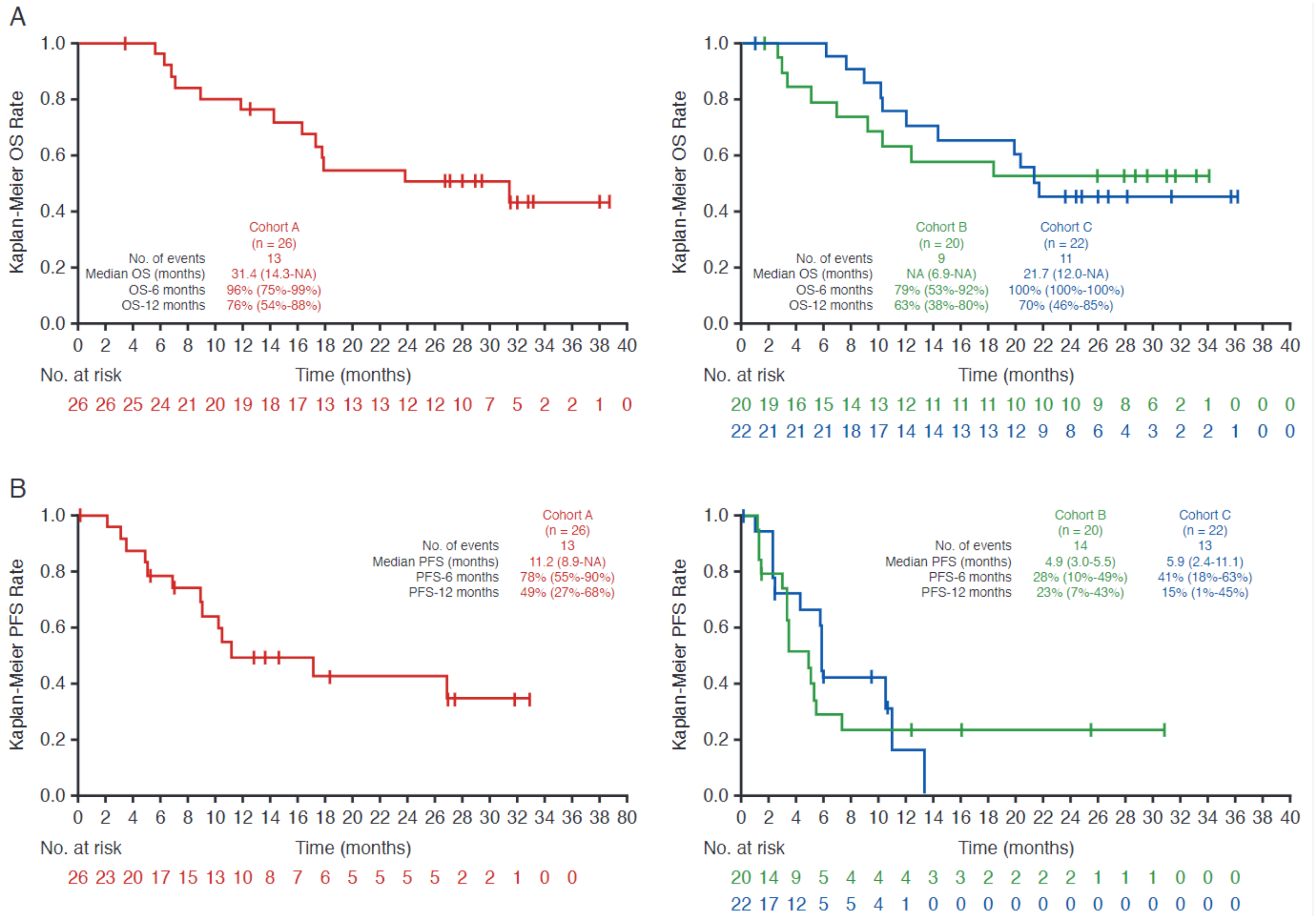

Cohort A, n=26; Cohort B, n=20; Cohort C, n=22

NA, not available; OS, overall survival; PFS, progression-free survival.

Supplementary figure 3. Pearson correlation was quantified across all RNAseq samples, and samples were ordered using hierarchical clustering with average agglomeration. Samples with very low concordance (average r = 0.07–0.24) were removed; these are clearly seen by the white-ish bars along the x and y axes.

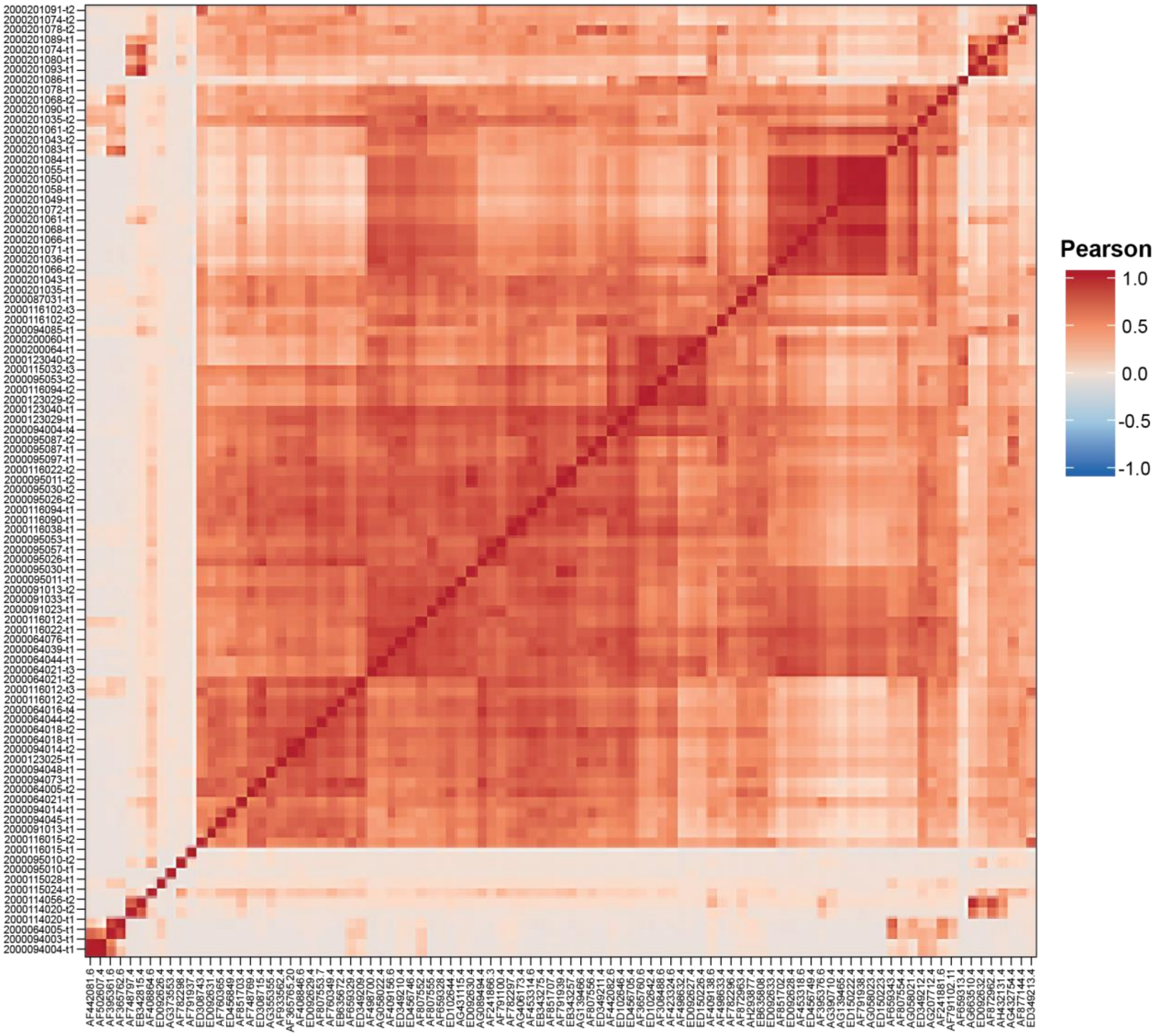

Supplementary figure 4. Summary of patients with RNAseq data correlates. The strata (colors) are associated with the patient cohort, and the numbers of patients associated with each category are indicated on the y axis.

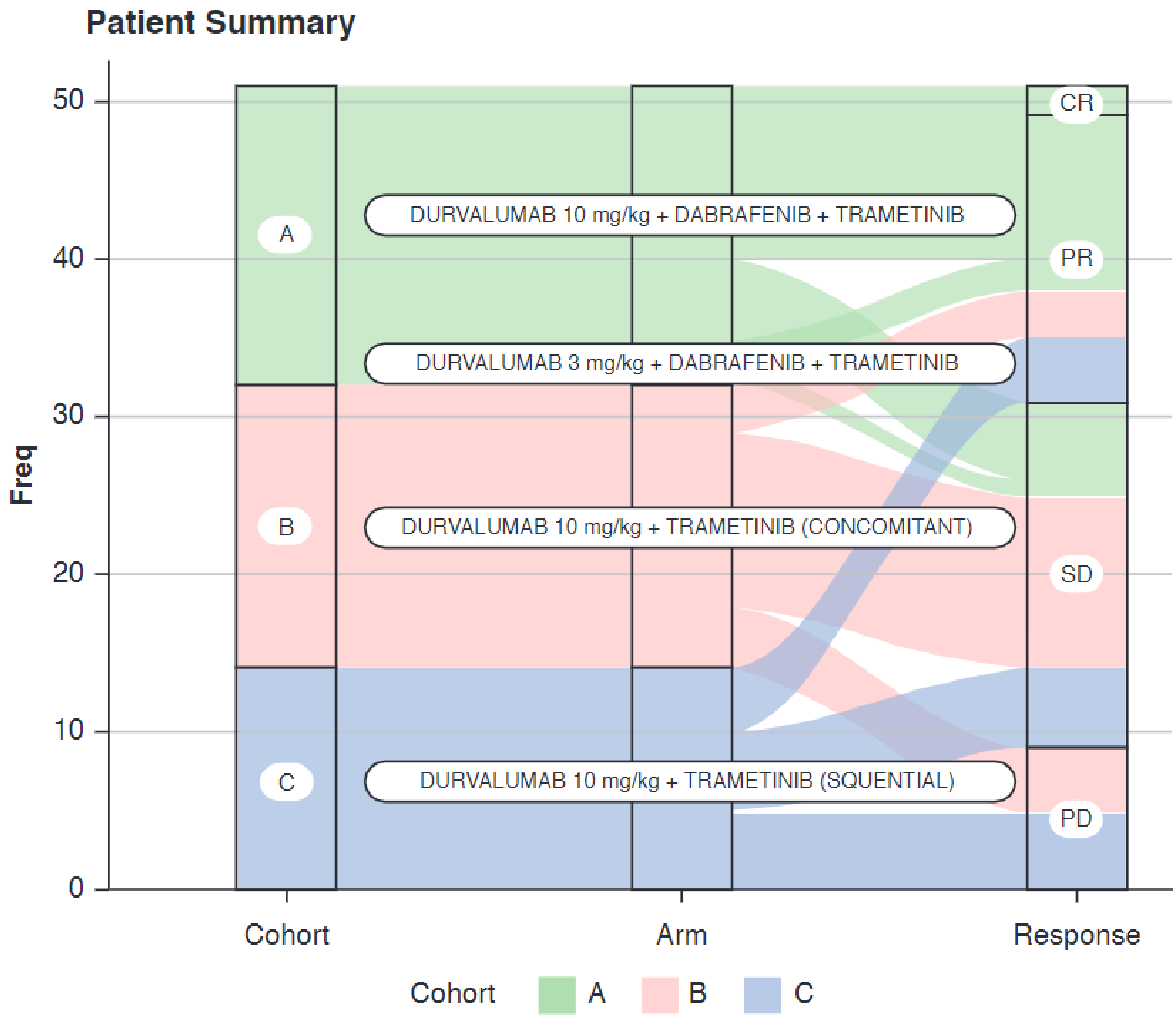

Freq, frequency

Supplementary figure 5. Study diagram.

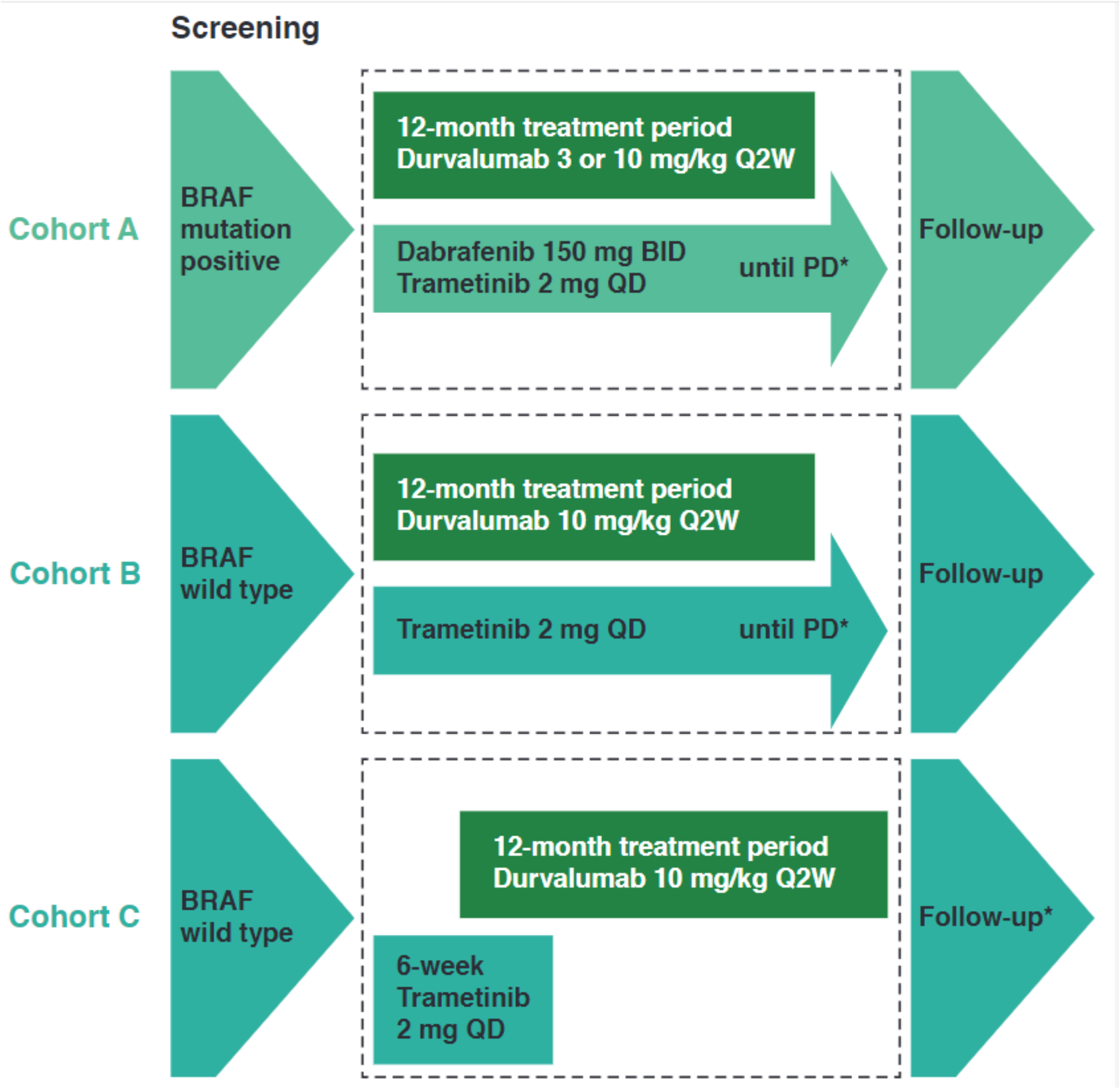

*If patients had disease progression after durvalumab treatment, it could be restarted alone or in addition to trametinib and/or dabrafenib for an additional 12 months or until confirmed disease progression. For cohort C, durvalumab could be reinitiated for an additional 12 months in the absence of trametinib. Each of the 3 cohorts was expanded to further evaluate the safety and antitumor activity of durvalumab in combination with dabrafenib and trametinib or with trametinib alone. The follow-up period included visits at 30, 60, and 90 days after the end of treatment and then monthly or bimonthly visits or telephone calls as appropriate.

Dose escalation followed a standard 3+3 design. At least 3 patients were enrolled in each dose cohort. If no more than 1 of the 3 patients in any dose cohort experienced a DLT, the dose cohort was expanded to a minimum of 6 patients. If no more than 1 of these 6 patients experienced a DLT, the dose escalation could proceed. If 2 or more patients in a dose cohort experienced a DLT, the MTD was deemed to be exceeded and no further patients were enrolled in that cohort.

BID, twice daily; PD, progressive disease; Q2W, every 2 weeks; QD, once daily.

Supplementary Tables

Supplementary table 1. Most frequent treatment-emergent adverse events related to any of the study drugs (≥20% of patients in any cohort) (as-treated population)

Preferred term, n (%)[a]	Cohort A: Durvalumab 3 or 10 mg/kg + dabrafenib + trametinib (n=26)		Cohort B: Durvalumab 10 mg/kg + trametinib (concurrent) (n=20)		Cohort C: Durvalumab 10 mg/kg + trametinib (sequential) (n=22)	
	**Any grade**	**Grade 3/4**	**Any grade**	**Grade 3/4**	**Any grade**	**Grade 3/4**
**Diarrhea**	12 (46.2)	2 (7.7)	11 (55.0)	0	9 (40.9)	1 (4.5)
**Fatigue**	16 (61.5)	0	7 (35.0)	1 (5.0)	6 (27.3)	0
**Rash**	9 (34.6)	1 (3.8)	7 (35.0)	0	11 (50.0)	2 (9.1)
**Pyrexia**	20 (76.9)	2 (7.7)	2 (10.0)	0	2 (9.1)	1 (4.5)
**Chills**	17 (65.4)	0	1 (5.0)	0	3 (13.6)	0
**Vomiting**	11 (4.23)	1 (3.8)	1 (5.0)	0	8 (36.4)	0
**Peripheral edema**	6 (23.1)	0	4 (20.0)	0	9 (40.9)	0
**Nausea**	11 (42.3)	0	1 (5.0)	0	6 (27.3)	0
**Dermatitis acneiform**	6 (23.1)	0	6 (30.0)	0	5 (22.7)	0
**Arthralgia**	13 (50.0)	0	0	0	3 (13.6)	0
**Asthenia**	7 (26.9)	0	5 (25.0)	0	3 (13.6)	1 (4.5)
**Blood creatine phosphokinase increased**	4 (15.4)	1 (3.8)	5 (25.0)	1 (5.0)	6 (27.3)	3 (13.6)
**Dry mouth**	9 (36.4)	0	2 (10.0)	0	4 (18.2)	0
**Pruritus**	3 (11.5)	0	2 (10.0)	0	9 (40.9)	0
**Decreased appetite**	6 (23.1)	0	3 (15.0)	0	4 (18.2)	0
**Folliculitis**	2 (7.7)	0	5 (25.0)	1 (5.0)	6 (27.3)	0
**Aspartate aminotransferase increased**	6 (23.1)	2 (7.7)	2 (10.0)	0	2 (9.1)	0
**Rash maculo-papular**	7 (26.9)	1 (3.8)	2 (10.0)	1 (5.0)	1 (4.5)	0
**Alanine aminotransferase increased**	6 (23.1)	1 (3.8)	2 (10.0)	0	1 (4.5)	0
**Headache**	6 (23.1)	0	0	0	2 (9.1)	0
**Hyperhidrosis**	8 (30.8)	0	0	0	0	0

[a]Patients are counted once for each preferred term, regardless of the number of events.

Supplementary table 2. RNA-sequencing sample exclusions

All samples were analyzed for quality of RNA sequencing; 11 out of 95 samples had very low concordance with the rest of the cohort (see **Figure 1**).

Sample ID	ID	Cohort	Visit	Site	Response
AF408864-6	2000095010	A	Day 15	Lung	PR
ED092626-4	2000201071	A	Screening	Lymph Node	PR
AG373553-4	2000095053	A	Day 15	Liver	SD
AF364762-6	2000114020	B	Day 15	Lymph Node	SD
AF502607-4	2000094014	B	Day 15	Skin	PD
AF91937-4	2000091023	C	Screening	Liver	PR
AF749797-4	2000116012	C	Day 43	Lymph Node	PD

Supplementary table 3. Geometric mean (n, geometric CV%) of durvalumab serum concentration (µg/mL) (PK-evaluable population)

	**Cohort A**		**Cohort B**	**Cohort C**
	**Durvalumab 3 mg/kg + dabrafenib + trametinib (n=6)**	**Durvalumab 10 mg/kg + dabrafenib + trametinib (n=20)**	**Durvalumab 10 mg/kg + trametinib (concurrent) (n=20)**	**Durvalumab 10 mg/kg + trametinib (sequential) (n=22)**
$C_{max, 1st}$	55.19 (6, 22.3)	238.44 (19, 23.1)	241.51 (19, 31.8)	266.79 (16, 59.5)
$C_{trough, ss}$	41.83 (4, 57.7)	150.51 (16, 66.7)	162.83 (10, 36.8)	176.37 (18, 87.5)
$C_{max, ss}$	82.86 (4, 21.4)	395.22 (6, 26.1)	427.56 (2, 23.3)	478.27 (6, 37.6)

$C_{max,1st}$, end of infusion on Day 1 for Cohorts A and B; end of infusion on Day 29 for Cohort C.

$C_{trough,ss}$, Pre-dose on Day 141 for Cohorts A and B; pre-dose on Day 169 for Cohort C.

$C_{max,ss}$, End of infusion on Day 141 for Cohorts A and B; end of infusion on Day 169 for Cohort C.

$C_{max,1st}$, peak concentration after first dose; $C_{max,ss}$, peak concentration at steady state; $C_{trough,ss}$, trough concentration at steady state; CV%, coefficient of variation; PK, pharmacokinetic(s)

Supplementary table 4. Geometric mean (n, geometric CV%) of dabrafenib, desmethyl-dabrafenib, and hydroxy-dabrafenib pre-dose plasma concentration (µg/mL) (PK-evaluable population)

	**Dabrafenib**		**Desmethyl-dabrafenib**		**Hydroxy-dabrafenib**	
	**Durvalumab 3 mg/kg + dabrafenib + trametinib (n=6)**	**Durvalumab 10 mg/kg + dabrafenib + trametinib (n=20)**	**Durvalumab 3 mg/kg + dabrafenib + trametinib (n=6)**	**Durvalumab 10 mg/kg + dabrafenib + trametinib (n=20)**	**Durvalumab 3 mg/kg + dabrafenib + trametinib (n=6)**	**Durvalumab 10 mg/kg + dabrafenib + trametinib (n=20)**
Day 15, pre-dose	0.1859 (4, 154.0)	0.1374 (18, 356.5)	0.2481 (4, 76.2)	0.2458 (19, 223.1)	0.1162 (4, 164.0)	0.1089 (18, 236.0)
Day 29, pre-dose	0.0462 (4, 100.7)	0.1079 (14, 313.8)	0.2574 (4, 47.3)	0.3263 (14, 89.1)	0.0408 (4, 156.8)	0.0873 (14, 202.4)
Day 43, pre-dose	0.0421 (4, 248.7)	0.0665 (15, 356.2)	0.2057 (4, 24.2)	0.2267 (15, 54.8)	0.0346 (4, 217.6)	0.0662 (15, 180.7)
Day 71, pre-dose	0.0696 (4, 393.9)	0.0845 (16, 643.4)	0.3756 (4, 28.4)	0.2529 (16, 85.0)	0.0773 (4, 240.4)	0.0780 (16, 320.2)

CV%, coefficient of variation; PK, pharmacokinetic(s)

Supplementary table 5. Geometric mean (n, geometric CV%) of trametinib pre-dose plasma concentration (µg/mL) (PK-evaluable population)

	Cohort A		Cohort B	Cohort C
	**Durvalumab 3 mg/kg + dabrafenib + trametinib (n=6)**	**Durvalumab 10 mg/kg + dabrafenib + trametinib (n=20)**	**Durvalumab 10 mg/kg + trametinib (concurrent) (n=20)**	**Durvalumab 10 mg/kg + trametinib (sequential) (n=22)**
Day 15, pre-dose	0.0126 (4, 38.9)	0.0123 (19, 55.8)	0.0143 (20, 64.3)	0.0119 (17, 67.6)
Day 29, pre-dose	0.0055 (5, 104.7)	0.0105 (16, 60.6)	0.0071 (20, 154.5)	0.0096 (22, 158.1)
Day 43, pre-dose	0.0065 (4, 59.6)	0.0084 (17, 115.6)	0.0052 (17, 262.4)	0.0105 (4, 39.9)
Day 71, pre-dose	0.0097 (4, 33.6)	0.0083 (18, 160.2)	0.0083 (11, 203.9)	-

CV%, coefficient of variation; PK, pharmacokinetic(s)

Supplementary table 6. List of institutions where the ethical committee approved the study protocol

Region	Site #	Site IRB Types	Site Name	IRB Account Name
CANADA	2000095	Local	Princess Margaret Hospital - Toronto	University Health Network Research Ethics Board
CANADA	2000116	Local	Sir Mortimer B. Davis Jewish General Hospital	Sir Mortimer B. Davis, Jewish General Hospital, Research Ethics Committee
FRANCE	2000201	Local	Centre Institut Gustave Roussy	Comité de Protection des Personnes Ile de France III
ITALY	2000087	Central	Istituto Nazionale Tumori IRCCS Fondazione Pascale	Comitato Etico Istituto Nazionale per lo Studio e la Cura dei Tumori Fondazione G. Pascale
UNITED KINGDOM	2000169	Central	East and North Hertfordshire NHS Trust	Research Ethics Committee - London Central
UNITED STATES	2000064	Local	University of California Los Angeles	Office of the Human Research Protection Program (OHRPP)
UNITED STATES	2000091	Local	Massachusetts General Hospital	Dana Farber Cancer Institute - Office for Human Research Studies
UNITED STATES	2000091	Local	Massachusetts General Hospital	Dana-Farber Cancer Institute Institutional Review Board
UNITED STATES	2000094	Local	Mount Sinai Medical Center Comprehensive Cancer Center	Mount Sinai Medical Center Institutional Review Board
UNITED STATES	2000114	Local	HonorHealth Research Institute	Western Institutional Review Board
UNITED STATES	2000115	Local	Northwestern Medical Faculty Foundation	Northwestern University Institutional Review Board
UNITED STATES	2000123	Local	Washington University School of Medicine Siteman Cancer Center	Washington University School of Medicine, Human Studies Committee
UNITED STATES	2000131	Local	Providence Portland Medical Center/Providence Cancer Center	Providence Health & Services Oregon and Southwest Washington Institutional Review Board
UNITED STATES	2000142	Local	Memorial Sloan-Kettering Cancer Center	Memorial Sloan-Kettering Cancer Center, Institutional Review Board
UNITED STATES	2000200	Local	University of California San Francisco	Committee on Human Research
