# Supplementary material for: PD-L1 blockade in combination with inhibition of MAPK oncogenic signaling in patients with advanced melanoma
Source: Nat Commun. 2020 Dec 7;11:6262. doi: 10.1038/s41467-020-19810-w (PMC7721806; doi:10.1038/s41467-020-19810-w)
Supplement: Supplementary file 2 — Reporting Summary [file 41467_2020_19810_MOESM2_ESM.pdf]

nature research

Corresponding author(s): Antoni Ribas

Last updated by author(s): Oct 15, 2020

Reporting Summary

Nature Research wishes to improve the reproducibility of the work that we publish. This form provides structure for consistency and transparency in reporting. For further information on Nature Research policies, see Authors & Referees and the Editorial Policy Checklist .

Statistics

For all statistical analyses, confirm that the following items are present in the figure legend, table legend, main text, or Methods section.

n/a	Confirmed	
☐	☒	The exact sample size (*n*) for each experimental group/condition, given as a discrete number and unit of measurement
☒	☐	A statement on whether measurements were taken from distinct samples or whether the same sample was measured repeatedly
☐	☒	The statistical test(s) used AND whether they are one- or two-sided<br>*Only common tests should be described solely by name; describe more complex techniques in the Methods section.*
☐	☒	A description of all covariates tested
☒	☐	A description of any assumptions or corrections, such as tests of normality and adjustment for multiple comparisons
☐	☒	A full description of the statistical parameters including central tendency (e.g. means) or other basic estimates (e.g. regression coefficient) AND variation (e.g. standard deviation) or associated estimates of uncertainty (e.g. confidence intervals)
☐	☒	For null hypothesis testing, the test statistic (e.g. *F*, *t*, *r*) with confidence intervals, effect sizes, degrees of freedom and *P* value noted<br>*Give P values as exact values whenever suitable.*
☒	☐	For Bayesian analysis, information on the choice of priors and Markov chain Monte Carlo settings
☐	☒	For hierarchical and complex designs, identification of the appropriate level for tests and full reporting of outcomes
☐	☒	Estimates of effect sizes (e.g. Cohen's *d*, Pearson's *r*), indicating how they were calculated

Our web collection on statistics for biologists contains articles on many of the points above.

Software and code

Policy information about availability of computer code

Data collection	For the RNA sequencing (RNAseq), total RNA from each sample was prepared for sequencing using the Takara Bio SMART-Seq: SMART-Seq® v4 Ultra® Low Input RNA Kit. Between 0.8–1.3ng of RNA was used to prepare the RNA libraries. Eleven cycles of PCR were performed during cDNA amplification. Samples were then processed with the Nextera XT DNA sample preparation kits for Illumina. The cDNA was normalized to the modified recommended input amount of 100–150pg and ten cycles during Nextera library prep. The purified amplified libraries were then validated by Agilent High Sensitivity DNA chip on Agilent 2100 Bioanalyzer and quantitated via qPCR using KAPA Library Quantification Kit (KAPA Biosystems) according to manufacturer's instructions. The libraries were sequenced on Illumina HiSeq 2500 with the following run parameters: Paired-End/Dual-Indexed 2×75bp reads.
Data analysis	RNAseq data was aligned to the human reference genome (GRCh38) by Hisat2 (version 2.0.4). Gene expression was annotated using Ensembl (release 94) and summarized by HTSeq-counts (version 0.6.1). Gene expression values were normalized and compared across groups using the DESeq2 (version 1.28.1) R package (version 3.0.0). Gene expression was displayed as the z-score of the normalized gene expression using the ggplot2 (version 3.3.2) R package (version 3.0.0).

For manuscripts utilizing custom algorithms or software that are central to the research but not yet described in published literature, software must be made available to editors/reviewers. We strongly encourage code deposition in a community repository (e.g. GitHub). See the Nature Research guidelines for submitting code & software for further information.

Data

Policy information about availability of data

All manuscripts must include a data availability statement. This statement should provide the following information, where applicable:

- Accession codes, unique identifiers, or web links for publicly available datasets
- A list of figures that have associated raw data
- A description of any restrictions on data availability

The clinical dataset analyzed here is available and may be obtained in accordance with AstraZeneca's data sharing policy, which is described at https://astrazenecagrouptrials.pharmacm.com/ST/Submission/Disclosure. RNAseq data is available in GEO under GSE158403 [https://www.ncbi.nlm.nih.gov/geo/query/

acc.cgi?acc=GSE158403].

Field-specific reporting

Please select the one below that is the best fit for your research. If you are not sure, read the appropriate sections before making your selection.

- [x] Life sciences
- [ ] Behavioural & social sciences
- [ ] Ecological, evolutionary & environmental sciences

For a reference copy of the document with all sections, see nature.com/documents/nr-reporting-summary-flat.pdf

Life sciences study design

All studies must disclose on these points even when the disclosure is negative.

Sample size

A total of up to 69 patients were required for both the dose-escalation phase and the dose-expansion phase of the study. For the dose-escalation phase, up to 24 evaluable patients were required, with 2 dose
levels in Cohort A and 1 dose level each in Cohorts B and C. For the dose-expansion phase, a total of 42 subjects were needed in 3 expansion cohorts consisting of approximately 14 subjects in each of Cohorts A, B, and C. The goal was to have ~20 subjects in each cohort treated at the MTD or 10 mg/kg dose level selected for each cohort. The sample size was primarily chosen to obtain preliminary assessment of antitumor activity (ORR).

Data exclusions

No data were excluded from these analyses.

Replication

This article reports on a clinical trial and correlative studies in patient-derived biopsies. The clinical trial and the planning for sample analyses were conducted after prospective planning included in the clinical trial protocol.

Randomization

Study participation began once written informed consent was obtained, and a subject identification (SID) number was assigned by a central system (Parexel). Once study eligibility (inclusion/exclusion) was assessed, the SID number was used to identify the subject during the screening process and throughout study participation. Patients were randomized to received either durvalumab in combination with dabrafenib and trametinib or with trametinib alone.

Blinding

This was a Phase I, open-label study, therefore the study was not blinded. Each subject who met the eligibility criteria was assigned to a treatment arm.

Reporting for specific materials, systems and methods

We require information from authors about some types of materials, experimental systems and methods used in many studies. Here, indicate whether each material, system or method listed is relevant to your study. If you are not sure if a list item applies to your research, read the appropriate section before selecting a response.

Materials & experimental systems

n/a	Involved in the study	
[ ]	[x]	Antibodies
[x]	[ ]	Eukaryotic cell lines
[x]	[ ]	Palaeontology
[x]	[ ]	Animals and other organisms
[ ]	[x]	Human research participants
[ ]	[x]	Clinical data

Methods

n/a	Involved in the study	
[x]	[ ]	ChIP-seq
[x]	[ ]	Flow cytometry
[x]	[ ]	MRI-based neuroimaging

Antibodies

Antibodies used

Antibodies used were as follows; MEDI4736 200mg Lyophilized Powder, 10R vial (DP lot number - BF0093; IMP lot number - MEDI-00875); MEDI4736 200mg Lyophilized Powder, 10R vial (DP lot number - BL0073; IMP lot number - MEDI-00939); MEDI4736 200mg Lyophilized Powder, 10R vial (DP lot number - CJ0035; IMP lot number - MEDI-01346)

Validation

All immunoassays included in the study had been previously validated.

Human research participants

Policy information about studies involving human research participants

Population characteristics	Adult patients with stage IIIC/IV melanoma (BRAFV600-mutant or BRAF-wild type metastatic/unresectable) were eligible for inclusion. Patients were required to have ≥ 1 measurable lesion per Response Evaluation Criteria in Solid Tumors (RECIST) version 1.131, an Eastern Cooperative Oncology Group performance status of 0 or 1, and adequate bone marrow and organ function. Prior immunotherapy with anti–CTLA-4 or anti–PD-1/PD-L1 agent was permitted.
Recruitment	The sites used their existing patient population to determine which subjects would meet the inclusion/exclusion criteria and then invited them to review the informed consent form. Subjects at multiple sites were screened for each cohort to ensure enrollment was competitive and cohorts were balanced.
Ethics oversight	1. Princess Margaret Hospital - Toronto University Health Network Research Ethics Board<br>2. Sir Mortimer B. Davis Jewish General Hospital Sir Mortimer B. Davis, Jewish General Hospital, Research Ethics Committee<br>3. Centre Institut Gustave Roussy Comité de Protection des Personnes Ile de France III<br>4. Istituto Nazionale Tumori IRCCS Fondazione Pascale Comitato Etico Istituto Nazionale per lo Studio e la Cura dei Tumori Fondazione G. Pascale<br>5. East and North Hertfordshire NHS Trust Research Ethics Committee - London Central<br>6. University of California Los Angeles Office of the Human Research Protection Program (OHRPP)<br>7. Massachusetts General Hospital Dana Farber Cancer Institute - Office for Human Research Studies<br>8. Massachusetts General Hospital Dana-Farber Cancer Institute Institutional Review Board<br>9. Mount Sinai Medical Center Comprehensive Cancer Center Mount Sinai Medical Center Institutional Review Board<br>10. HonorHealth Research Institute Western IRB<br>11. Northwestern Medical Faculty Foundation Northwestern University Institutional Review Board<br>12. Washington University School of Medicine Siteman Cancer Center Washington University School of Medicine, Human Studies Committee<br>13. Providence Portland Medical Center/Providence Cancer Center Providence Health & Services Oregon and Southwest Washington Institutional Review Board<br>14. Memorial Sloan-Kettering Cancer Center Memorial Sloan-Kettering Cancer Center, Institutional Review Board<br>15. University of California San Francisco Committee on Human Research

Note that full information on the approval of the study protocol must also be provided in the manuscript.

Clinical data

Policy information about clinical studies

All manuscripts should comply with the ICMJEguidelines for publication of clinical research and a completedCONSORT checklist must be included with all submissions.

Clinical trial registration	NCT02027961
Study protocol	The study protocol can be found in the Supplementary Notes; Section 4.5 of the study protocol detailing the formulation of durvalumab has been redacted due to legal/intellectual property requirements set by the manufacturer.
Data collection	Data collection took place at the assigned study site. A Web Based Data Capture system was used to collect data. The investigator was responsible for ensuring data were recorded on the eCRFs as specified in the study protocol. The investigator monitored the accuracy, completeness, and timeliness of the data recorded and ensured the provision of answers to data queries were according to the Clinical Study Agreement. The first patient was enrolled on 20 December 2013 and the last visit for the final patient was on 24 April 2018. Data cutoff for this analysis was May 30, 2018, when there was at least three years of follow up in all study subjects.
Outcomes	The primary objective of this study was to determine the MTD and to characterize the safety profile of durvalumab in combination with dabrafenib and trametinib or with trametinib alone in subjects with metastatic or unresectable melanoma with BRAF mutation positive or WT BRAF, respectively, as assessed by measuring DLTs, AEs, SAEs, laboratory evaluations, vital signs, physical examinations and ECHO/ECG results.<br>A secondary objective was to evaluate the antitumor activity of durvalumab in combination with dabrafenib and trametinib in BRAF mutation-positive melanoma and BRAF WT melanoma, as measured by OR, DC, DoR, PFS, and OS. An additional secondary objective was to describe the PK of durvalumab in combination with dabrafenib and trametinib or with trametinib alone by measuring individual durvalumab concentrations in serum and PK parameters. A final secondary objective was to determine the immunogenicity of durvalumab with dabrafenib and trametinib or with trametinib alone as measured by the number and percentage of subjects who developed detectable ADAs.
