# Supplementary material for: PD-L1 blockade in combination with inhibition of MAPK oncogenic signaling in patients with advanced melanoma
Source: Nat Commun. 2020 Dec 7;11:6262. doi: 10.1038/s41467-020-19810-w (PMC7721806; doi:10.1038/s41467-020-19810-w)
Supplement: Supplementary file 4 — Description of Additional Supplementary File [file 41467_2020_19810_MOESM4_ESM.pdf]

Description of Additional Supplementary File

File Name: Supplementary Data 1
Description: Patient characteristics and bioinformatics data
